# Differentiable samplers for deep latent variable models

**DOI:** 10.1098/rsta.2022.0147

**Published:** 2023-05-15

**Authors:** Arnaud Doucet, Eric Moulines, Achille Thin

**Affiliations:** ^1^ Department of Statistics, Oxford University, Oxford, UK; ^2^ Ecole Polytechnique, Centre de Mathématiques Appliquées, CNRS UMR 7641, Palaiseau, France

**Keywords:** Bayesian inference, importance sampling, Monte Carlo methods, variational inference

## Abstract

Latent variable models are a popular class of models in statistics. Combined with neural networks to improve their expressivity, the resulting deep latent variable models have also found numerous applications in machine learning. A drawback of these models is that their likelihood function is intractable so approximations have to be carried out to perform inference. A standard approach consists of maximizing instead an evidence lower bound (ELBO) obtained based on a variational approximation of the posterior distribution of the latent variables. The standard ELBO can, however, be a very loose bound if the variational family is not rich enough. A generic strategy to tighten such bounds is to rely on an unbiased low-variance Monte Carlo estimate of the evidence. We review here some recent importance sampling, Markov chain Monte Carlo and sequential Monte Carlo strategies that have been proposed to achieve this.

This article is part of the theme issue ‘Bayesian inference: challenges, perspectives, and prospects’.

## 1. Latent variable models

We consider here that observation $x\in X\subset R^{d_{x}}$ is a sample from a probability density $p^{*}(x)$. We approximate $p^{*}(x)$ by considering a parametric model $p_{\theta }(x)$, with parameters $\theta \in \Theta \subset R^{d}$, defined by

1.1 $$p_{\theta }(x)=\int_{Z}p_{\theta }(x,z)\;\text{d}z,$$

where $z\in Z\subset R^{d_{z}}$ is a latent variable. Such an implicit distribution over x is very flexible. If z is discrete and $p_{\theta }(x|z)$ is a Gaussian distribution, then $p_{\theta }(x)$ is a Gaussian mixture. If z is continuous as considered here, $p_{\theta }(x)$ is an infinite mixture of the $p_{\theta }(x|z)$, thus more expressive than finite mixtures. We use the term deep latent variable model (DLVM) to refer to a latent variable model $p_{\theta }(x,z)$ whose distribution is parameterized by neural networks. DLVMs include, in particular, the deep latent Gaussian models (DLGMs) [1]—which encompass the popular *variational auto-encoder* [2–4] described further. They have a wide range of applications, from generative modelling to semi-supervised learning and representation learning.

DLGMs rely on $p_{\theta }(x,z)=p(z)p_{\theta }(x|z)$, where p(z) is a standard multivariate Gaussian prior distribution over latent space and $p_{\theta }(x|z)$ is the conditional distribution of the observation given the latent variables (also referred to as the *decoder*). An advantage of DLGMs and more generally DLVMs is that the marginal distribution $p_{\theta }(x)$ can be very complex, even if each factor (prior and decoder) in the model is relatively simple. When considered as a function $\theta \mapsto p_{\theta }(x)$ for a given x, $p_{\theta }(x)$ is called the *marginal likelihood* or *evidence*—we sometimes omit ‘marginal’, when there is no ambiguity. For a given value of x, $p_{\theta }(x)$ is the normalizing constant of the unnormalized distribution $z\to p_{\theta }(x,z)$.

Given data x, we would like to maximize the log marginal likelihood $\ell (\theta ,x)=\log p_{\theta }(x)$. The main difficulty of maximum-likelihood learning in complex latent variable models such as DLVMs is that ℓ(θ,x) is intractable. This intractability is due to the integral (1.1) which must be computed to obtain the model evidence. Because of this intractability, we cannot easily differentiate and optimize these models according to their parameters. While Fisher’s identity shows that

1.2 $$\nabla_{\theta }\ell (\theta ,x)=\int \nabla \log p_{\theta }(x,z)\;p_{\theta }(z|x)\;\text{d}z,$$

the posterior distribution $p_{\theta }(z|x)$ is itself intractable and importance sampling or Markov chain Monte Carlo (MCMC) approximations of it are typically necessary.

The approach that has become prominent in the machine learning literature is to rely instead on variational inference (VI). VI introduces a parametric family of distributions, the variational family $Q=\{q_{\phi },\phi \in \Phi \}$, which is parameterized by $\phi \in \Phi \subset R^{d^{\prime }}$. The goal is to choose the parameter ϕ such that $q_{\phi }(z|x)$ approximates the true posterior $p_{\theta }(z|x)$ for given x,θ, typically intractable for DLVMs. The likelihood function is defined via a generally intractable integral. To circumvent marginalization, a common approach is to optimize a *variational lower bound* on the log marginal likelihood (often abusively referred to as the marginal log-likelihood); see [2,5,6].

1.3 $$L(\theta ,\phi )=\int_{Z}\log\left(\frac{p_{\theta }(x,z)}{q_{\phi }(z|x)}\right)q_{\phi }(z|x)\;\text{d}z=\ell (\theta ,x)-D_{\text{KL}}(q_{\phi }(\cdot |x)||p_{\theta }(\cdot |x)),$$

where $D_{\text{KL}}$ denotes the Kullback–Leibler divergence. This objective is thus always lower than the marginal log-likelihood ℓ(θ,x) and is thus referred to as the evidence lower bound (ELBO). The expressiveness of Q is essential for good performance; e.g. [7]. The maximization of the ELBO implicitly solves two optimization problems concurrently: (i) maximizing the log evidence $\log p_{\theta }(x)$ and (ii) minimizing the divergence $D_{\text{KL}}(q_{\phi }(\cdot |x)||p_{\theta }(\cdot |x))$ between the variational posterior and the true posterior. *Amortized variational inference* [2] proposes to learn one global mapping in x which maps each observation to a distribution in the latent space that approximates $z\mapsto p_{\theta }(z|x)$. The parameters ϕ are thus shared among all the distributions $\left\{q_{\phi }(\cdot |x_{1}),\ldots ,q_{\phi }(\cdot |x_{N})\right\}$. This is useful as practically we typically have a large dataset $x_{1},\ldots ,x_{N}$ and the resulting ELBO is a sum of N terms.

If the density of $q_{\phi }(\cdot |x)$ is available in closed form, then the Q is called explicit. Explicit models allow straightforward estimation of the VI target, but often result in reduced flexibility, which limits their overall performance. For example, the mean-field VI [8] requires restrictive independence assumptions among the latent variables of interest: $Q=\{q_{\phi }(\cdot |x)=N(\mu_{\phi }(x),\text{diag}(\sigma^{2}(x)))\}$, where N(μ,Σ) denotes the d-dimensional normal distribution with mean μ and covariance Σ. In that case, the model is the very popular variational autoencoder (VAE) [2–4]. Normalizing flows (NFs) [1,^4^,^9^,^11^] provide an alternative family of explicit density models that have improved power compared with mean-field alternatives. These methods push samples from a simple base distribution (typically Gaussian) through parameterized diffeomorphisms to produce complex density models. NFs have proven useful; e.g. [9,10,12,13] where flows have been shown to improve ELBO tightness. Although NFs can directly improve the expressiveness of mean-field VI schemes, their inherent bijectivity impose limitations, see [14–16].

We focus here on a complementary approach to obtain tight ELBOs that builds upon Monte Carlo methods and can be traced back to Mnih & Rezende [17]. This general framework is described in §2 where a basic importance sampling approach is introduced for illustration. We then explain how one can generalize this approach to state-of-the-art Monte Carlo estimates of the evidence including annealed importance sampling (AIS) and more general sequential importance sampling (SIS) schemes in §3. Finally, sequential Monte Carlo are discussed in §4.

## 2. Monte Carlo for variational inference

We first note that the standard ELBO (1.3) can be thought of as the expectation w.r.t. to $q_{\phi }(z|x)$ of a one sample unbiased importance sampling estimate of $p_{\theta }(x)$

2.1 $${\hat{p}}_{\theta }(x,z):=w_{\theta ,\phi }(z)\;\mathrm{and}\;w_{\theta ,\phi }(z)=\frac{p_{\theta }(x,z)}{q_{\phi }(z|x)}.$$

However, as pointed out by Mnih & Rezende [17], there is no need to limit ourselves to such an estimate. As long as we have access to a positive unbiased estimate ${\hat{p}}_{\theta }(x,u)$ of $p_{\theta }(x)$ where u represents all the random variables of density q(u)^1^ necessary to compute this estimate then we can also define an ELBO through

2.2 $$L(\theta ,\phi )=\int \log{\hat{p}}_{\theta }(x,u)\;q(u)\;\text{d}u\leq \log\left(\int {\hat{p}}_{\theta }(x,u)q(u)\;\text{d}u\right)=\ell (\theta ,x),$$

where we have used first Jensen’s inequality then the unbiasedness of the evidence estimate. This approach is interesting as a Taylor expansion shows that

2.3 $$L(\theta ,\phi )\approx \ell (\theta ,x)-\frac{1}{2}{\text{var}}_{q}\left[\frac{{\hat{p}}_{\theta }(x,u)}{p_{\theta }(x)}\right];$$

e.g. [16,18,19]. So if one can obtain an unbiased estimate of the evidence which has a small variance relative to $p_{\theta }{\left(x\right)}^{2}$, then the resulting ELBO will be tight.

As a first illustration of this principle, consider the case where we compute an estimate of the evidence using not one but K samples; i.e. $u=(z_{1},\ldots ,z_{k})$, $q(u)=\prod_{k=1}^{K}q_{\phi }(z_{k}|x)$ and

2.4 $${\hat{p}}_{\theta }(x,u):=\frac{1}{K}\sum_{k=1}^{K}w_{\theta ,\phi }(z_{k}).$$

The resulting ELBO (2.2) denoted $L_{\text{iw}}(\theta ,\phi )$ is known as the importance weighted ELBO (IW-ELBO). It was proposed by Burda *et al.* [20], who showed that it is monotonically increasing with K and converges asymptotically to ℓ(θ,x) under mild assumptions.

In this case, we have

2.5 $$\nabla_{\theta }L_{\text{iw}}(\theta ,\phi )=E_{q}\left[\sum_{k=1}^{K}{\bar{w}}_{\theta ,\phi }^{k}\nabla \log p_{\theta }(x,z_{k})\right]\;\mathrm{and}\;{\bar{w}}_{\theta ,\phi }^{k}=\frac{w_{\theta ,\phi }(z_{k})}{\sum_{l=1}^{K}w_{\theta ,\phi }(z_{l})}.$$

This shows that an unbiased gradient of this ELBO w.r.t. θ can be obtained by building a normalized importance sampling approximation ${\hat{p}}_{\theta }(z|x)=\sum_{k=1}^{K}{\bar{w}}_{\theta ,\phi }^{k}\delta_{z_{k}}$ of $p_{\theta }(z|x)$ and integrating $\nabla_{\theta }\log p_{\theta }(x,z)$ w.r.t. it. The exact same gradient estimator would be obtained by replacing ${\hat{p}}_{\theta }(z|x)$ in place of $p_{\theta }(z|x)$ in Fisher’s identity (1.2). This shows that the biased gradient approximation of the true log-likelihood ℓ(θ,x) obtained by importance sampling is nothing but an unbiased gradient of $L_{\text{iw}}(\theta ,\phi )$. The ELBO interpretation is more fruitful as it shows the exact objective one optimizes when using stochastic gradient ascent. Additionally, it also allows us to optimize the algorithmic parameter ϕ. However, standard gradient estimates of this ELBO for ϕ have poor properties as K increases due to the fact that $L_{\text{iw}}(\theta ,\phi )$ becomes then almost independent of ϕ (because of the convergence of the ELBO to the true log marginal likelihood), and thus decreasingly strongly the signal-to-noise ratio of the estimator of $\nabla_{\phi }L_{\text{iw}}(\theta ,\phi )$ [21]. One can remedy this behaviour by using a ‘sticking the landing’ variance reduction approach [22] or by doubly reparameterizing the gradient estimates of $\nabla_{\phi }L_{\text{iw}}(\theta ,\phi )$ [23].

In this section, we have presented a generic way to obtain an ELBO from any unbiased positive estimate of the evidence. However, we have so far limited ourselves practically to a basic importance sampling estimate of the evidence which performs poorly in high dimension. In the next sections, we discuss more sophisticated Monte Carlo techniques.

## 3. Annealed and sequential importance sampling

The problem of standard importance sampling techniques is that it is difficult to build a ‘good’ proposal distribution in high-dimensional spaces. A popular approach addressing this problem is AIS, an approach pioneered by Crooks [24] and Neal [25] based on early work by Jarzynski [26]. Despite having been introduced over 20 years ago, this approach remains one of the ‘gold standard’ techniques to estimate the evidence unbiasedly and can thus be used to define an ELBO [27,28]. However, as detailed below, it is difficult to obtain low-variance gradient estimators of this ELBO so a generalized version of the AIS estimate based on SIS [29,30] has been instead favoured [14–16,31,33].

For ease of presentation, we avoid here measure-theoretic notation but some of the Markov kernels discussed here do not necessarily admit a density w.r.t. Lebesgue measure; e.g. Metropolis–Hastings kernels admit an atomic component. We refer the reader to Thin *et al.* [27] for a rigorous presentation.

### (a) Evidence estimate using sequential importance sampling

The first ingredient of these techniques is to define a sequence of T+1 unnormalized density functions $\gamma_{0},\ldots ,\gamma_{T}$ bridging smoothly an easy-to-sample distribution $\gamma_{0}(z)=q_{\phi }(z|x)$ (the dependency in *x* is omitted to avoid overloading the notation) to the unnormalized target of interest $\gamma_{T}(z)=p_{\theta }(x,z)$. Typically one selects

3.1 $$\gamma_{t}(z)=q_{\phi }{\left(z|x\right)}^{1-\beta_{t}}p_{\theta }{\left(x,z\right)}^{\beta_{t}}\;\mathrm{and}\;\pi_{t}(z)=\frac{\gamma_{t}(z)}{\int \gamma_{t}(z^{\prime })\;\text{d}z^{\prime }},$$

for an annealing schedule $0=\beta_{0}<\cdots <\beta_{T}=1$. Typically, either the ${\left(\beta \right)}_{t=0}^{T}$ are learned, or following Grosse *et al.* [34], we can select them as $\beta_{t}={\tilde{\beta }}_{t}-{\tilde{\beta }}_{0}/({\tilde{\beta }}_{T}-{\tilde{\beta }}_{0})$, with ${\tilde{\beta }}_{t}=\sigma (\delta (2t-T))$, where δ can be fixed or optimized and σ is the sigmoid function. The second ingredient of these techniques is to define a sequence of forward Markov transition kernels $m_{t}(z_{t-1},z_{t})$, designed to “move” samples towards $\pi_{t}$. The most common choice is to select $m_{t}$ as a MCMC kernel leaving $\pi_{t}$ invariant. However, it is not necessary that $m_{t}$ be exactly invariant for $\pi_{t}$: it is perfectly possible to use a kernel that leaves $m_{t}$ ‘approximately’ invariant. As a result $m_{t}$ depends on θ,ϕ and x but this is not emphasized notationally for simplicity. Finally, we present here for simplicity our results for distributions admitting densities w.r.t. the Lebesgue measure, but the reasoning in itself does not depend on this assumption, see [16,27] for a more general presentation.

Based on these two ingredients, we can build the following proposal distribution on the path space $z_{0:T}\in Z^{T+1}$

3.2 $$\bar{q}(z_{0:T})=q_{\phi }(z_{0}|x)\prod_{t=1}^{T}m_{t}(z_{t-1},z_{t}).$$

By construction, the Tth marginal $q_{T}(z_{T})=\int \bar{q}(z_{0:T})\;\text{d}z_{0:T-1}$ is expected to be close to $p_{\theta }(z|x)$ for T large enough and MCMC kernels mixing fast enough. If we were able to evaluate this marginal density pointwise, then we could compute the following unbiased importance sampling estimate of the evidence

3.3 $$w_{\theta ,\phi }^{\text{mar}}(z_{T})=\frac{p_{\theta }(x,z_{T})}{q_{T}(z_{T})}.$$

However, the expression of this marginal density is generally not available. To bypass this issue, we introduce another unnormalized density on the path space

3.4 $$\bar{p}(x,z_{0:T})=p_{\theta }(x,z_{T})\prod_{t=0}^{T-1}l_{t}(z_{t+1},z_{t}),$$

where $l_{t}(z_{t+1},z_{t})$ is a sequence of auxiliary backward Markov transition kernels; i.e. $\int l_{t}(z_{t+1},z_{t})\;\text{d}z_{t}=1$. These kernels will also depend typically on θ,ϕ and x but this is omitted notationally. By construction, we thus have $\int {\bar{p}}_{\theta }(x,z_{0:T})\;\text{d}z_{0:T-1}=p_{\theta }(x,z_{T})$.

Having now defined both a proposal (3.2) and an extended target (3.4) on path space, we can define the following SIS estimate of the evidence [29,30]

3.5 $$w_{\theta ,\phi }^{\text{sis}}(z_{0:T})=\frac{\bar{p}(x,z_{0:T})}{\bar{q}(z_{0:T})}=\prod_{t=1}^{T}\frac{\gamma_{t}(z_{t})l_{t-1}(z_{t},z_{t-1})}{\gamma_{t-1}(z_{t-1})m_{t}(z_{t-1},z_{t})}.$$

This is a special case of the general framework introduced in §2 where $u=z_{0:T},q(u)=\bar{q}(z_{0:T})$ and ${\hat{p}}_{\theta }(x,u):=w_{\theta ,\phi }^{\text{sis}}(z_{0:T})$. As in §2, we can further average over N samples of $\bar{q}(z_{0:T})$.

### (b) Settings

In practice, we need to select appropriately the forward and backward kernels.

#### (i) Annealed importance sampling

The standard AIS approach makes the following choice. First one selects $m_{t}$ to be MCMC kernels of invariant distribution $\pi_{t}$; e.g. Metropolis–Hastings. Then we select the corresponding backward kernel $l_{t-1}$ as the reversal of $m_{t}$, i.e.

3.6 $$\pi_{t}(z_{t-1})m_{t}(z_{t-1},z_{t})=\pi_{t}(z_{t})l_{t-1}(z_{t},z_{t-1}),$$

so in particular we have $l_{t-1}=m_{t}$ if $m_{t}$ is reversible w.r.t. $\pi_{t}$. Implicitly, the reversal of $m_{t}$ means the kernel associated with the time-reversed process of that with transition dynamics given by $m_{t}$, that begins in stationarity. When (3.6) is satisfied, we can easily check that the evidence estimate equation (3.5) simplifies and becomes equal to

3.7 $$w_{\theta ,\phi }^{\text{ais}}(z_{0:T})=\prod_{t=1}^{T}\frac{\gamma_{t}(z_{t-1})}{\gamma_{t-1}(z_{t-1})}.$$

This estimate is elegant but it is unfortunately difficult to obtain a low-variance estimate of the gradient of this ELBO. This is due to the fact that the transition kernels $m_{t}$ will typically involve accept/reject steps.

Consider for example that $m_{t}$ is a Metropolis–Hastings type kernel. We follow here the derivation by Thin *et al.* [16]. If we use a reparameterized proposal then we can write

3.8 $$m_{t}(z_{t-1},z_{t})=\int n_{t}((z_{t-1},u),z_{t})g(u)\;\text{d}u,$$

where g(u) is typically a standard multivariate normal and

3.9 $$n_{t}((z_{t-1},u),z_{t})=\alpha_{t}(z_{t-1},u)\delta_{S_{t}(z_{t-1},u)}(z_{t})+\{1-\alpha_{t}(z_{t-1},u)\}\delta_{z_{t-1}}(z_{t}),$$

where $\delta_{z}(z^{\prime })$ denotes the Dirac delta function, and $S_{t}(z_{t-1},u)$ denotes the Metropolis–Hastings proposal from point $z_{t-1}$ with noise u (sampled from the distribution g). For example, if we consider $m_{t}(z_{t-1},z_{t})$ a random walk Metropolis algorithm of proposal $N(z^{\prime };z_{t-1},\sigma_{t}^{2}I)$ then we would have $S_{t}(z_{t-1},u)=z_{t-1}+\sigma_{t}u$ and $\alpha_{t}(z_{t-1},u)=\min(1,\gamma_{t}(S_{t}(z_{t-1},u))/\gamma_{t}(z_{t-1}))$, with g the standard multivariate normal. With the convention $\alpha_{t}(z_{t-1},u)=\alpha_{t}^{1}(z_{t-1},u),1-\alpha_{t}(z_{t-1},u)=\alpha_{t}^{0}(z_{t-1},u),S_{t}^{1}(z_{t-1},u)=S_{t}(z_{t-1},u)$ and $S_{t}^{0}(z_{t-1},u)=z_{t-1}$, we can thus rewrite

3.10 $$n_{t}((z_{t-1},u),z_{t})=\sum_{a_{t}=0}^{1}\alpha_{t}^{a_{t}}(z_{t-1},u)\delta_{S_{t}^{a_{t}}(z_{t-1},u)}(z_{t}).$$

Piecing the T steps together, it thus follows that the proposal in equation (3.2) can be summarized as first draw the noise for the T steps from $g(u_{1:T})=\prod_{t=1}^{T}g(u_{t})$, and sequentially for 1≤t≤T, (i) conditionally upon $u_{1:T}$, draw the Bernoulli r.v.s $a_{t}$ with $P(a_{t}=v|z_{0},u_{1:t-1},a_{1:t-1})=\alpha_{t}^{v}(z_{t-1},u_{t})$ for v∈{0,1} and, (ii) given $z_{0},u_{1:t},a_{1:t}$, compute the state $z_{t}$ given by

3.11 $$z_{t}=\Phi_{t}(z_{0},u_{1:t},a_{1:t})=S_{t}^{a_{t}}(z_{t-1},u_{t})=S_{t}^{a_{t}}(\Phi_{t-1}(z_{0},u_{1:t-1},a_{1:t-1}),u_{t})$$

with $\Phi_{0}(z_{0},u_{1:0},a_{1:0})=z_{0}$. Then, the joint distribution of the accept/reject binary random variables $a_{1:T}$ is given by $\beta (a_{1:T}|z_{0},u_{1:T})=\prod_{t=1}^{T}\alpha_{t}^{a_{t}}(\Phi_{t-1}(z_{0},u_{1:t-1},a_{1:t-1}),u_{t})$. The proposal $\bar{q}(z_{0:T})$ in equation (3.2) is thus equal to

3.12 $$\begin{array}{rl} & q_{\phi }(z_{0}|x)\int \cdots \int \sum_{a_{1:T}\in {\left\{0,1\right\}}^{T}}g(u_{1:T})\beta (a_{1:T}|z_{0},u_{1:T})\\ & \;\;\times \prod_{t=1}^{T}\delta_{S_{t}^{a_{t}}(\Phi_{t}(z_{0},u_{1:t-1},a_{1:t-1}),u_{t})}(x_{t})\;\text{d}u_{1:T}, \end{array}$$

and finally the ELBO corresponding to the AIS estimate in equation (3.7) can be rewritten as

3.13 $$L_{\text{ais}}=E_{\bar{q}(z_{0:T})}[\log w_{\theta ,\phi }^{\text{ais}}(z_{0:T})]=E_{q_{\phi }(z_{0}|x)g(u_{1:T})\beta (a_{1:T}|z_{0},u_{1:T})}[\log w_{\theta ,\phi }^{\text{ais}}(z_{0:T})],$$

where the states $z_{1:T}$ are fully deterministic functions of $z_{0},u_{1:T},a_{1:T}$, denoted $z_{1:T}=\Phi (z_{0},u_{1:T},a_{1:T})$. It follows that the gradient of this ELBO w.r.t. ϕ,θ is given by

3.14 $$\nabla L_{\text{ais}}=\nabla L_{\text{ais}}^{\left(1\right)}+\nabla L_{\text{ais}}^{\left(2\right)},$$

where

3.15 $$\begin{array}{rl} & \nabla L_{\text{ais}}^{\left(1\right)}=E[\nabla \log w_{\theta ,\phi }^{\text{ais}}(z_{0},\Phi (z_{0},u_{1:T},a_{1:T}))],\\ \;\mathrm{and}\; & \nabla L_{\text{ais}}^{\left(2\right)}=E[\nabla \log\beta (a_{1:T}|z_{0},u_{1:T})\cdot \log w_{\theta ,\phi }^{\text{ais}}(z_{0},\Phi (z_{0},u_{1:T},a_{1:T}))]. \end{array}\}$$

The first term $\nabla L_{\text{ais}}^{\left(1\right)}$ can typically be estimated with low variance but the second term $\nabla L_{\text{ais}}^{\left(2\right)}$ has a higher variance estimate due to the gradient estimate for the accept/reject discrete random variables $\nabla \log\beta (a_{1:T}|z_{0},u_{1:T})$ [35], see [27] for a further discussion. In [28], the second term is neglected, which introduces a bias that is difficult to quantify. In [16], a control variate technique inspired by Mnih & Rezende [17, Section 2.5.3] is developed to reduce the variance of the second term but it remains high.

#### (ii) Annealed importance sampling with unadjusted proposals

While the standard AIS estimate can be used to obtain an ELBO, we have seen that the resulting ELBO is difficult to optimize as AIS typically relies on MCMC kernels using accept/reject steps. To bypass this issue, it is possible instead to use unadjusted samplers such as the unadjusted Langevin algorithm (ULA) [16,28,36] and the unadjusted Hamiltonian algorithm (UHA) [28,31,33–37].

We start by considering the use of ULA transition kernels; i.e. we let

3.16 $$m_{t}(z_{t-1},z_{t})=N(z_{t};z_{t-1}+\delta \nabla \log\pi_{t}(z_{t-1}),2\delta \mathrm{Id}).$$

The resulting proposal equation (3.2) can thus be thought of as an Euler–Maruyama discretization of the following time-inhomogeneous Langevin diffusion on the time interval $\left[0,T_{0}\right]$ for $\delta =T_{0}/T$

3.17 $$\;\text{d}z_{s}=\nabla \log\pi_{s}(z_{s})\;\text{d}s+\sqrt{2}\;\text{d}B_{s},$$

where ${\left(B_{s}\right)}_{s\in [0,T_{0}]}$ is a multivariate Brownian motion. We slightly abuse notation here as $\pi_{t}$ in (3.16) corresponds to $\pi_{t\delta }$ in continuous time. Since the continuous-time homogeneous Langevin diffusion is known to be reversible, a reasonable choice for the backward kernel is to select $l_{t-1}=m_{t}$, as suggested in [27,28,36]. However, as $m_{t}$ is not exactly $\pi_{t}$-reversible, we need to rely on the general expression given in (3.5) for the evidence estimate. As this proposal can be easily reparameterized as a function of the Gaussian random variables used to sample from $m_{t}$, we can apply the reparameterization trick to compute low-variance gradient estimates of the corresponding ELBO. It was shown in [16] that, compared with a standard AIS estimate using Metropolis-adjusted Langevin kernels, the ELBO computed using ULA kernels was not as tight but it was much easier to optimize.

Instead of having a proposal arising from the time-discretization of an overdamped Langevin diffusion (3.17), we can instead build a proposal by discretizing an underdamped Langevin diffusion initialized at $z_{0}∼q_{\phi }(\cdot |x),p_{0}∼N(0,M)$

3.18 $$\;\text{d}x_{s}=M^{-1}p_{s}\;\text{d}s\;\mathrm{and}\;\;\text{d}p_{s}=\nabla \log\pi_{s}(z_{s})\;\text{d}s-\eta p_{s}\;\text{d}s+\sqrt{2\eta }M^{1/2}\;\text{d}B_{s}.$$

Here $p_{s}\in R^{d_{z}}$ is a momentum variable, M a positive definite mass matrix and η>0 a friction coefficient. If we had $\pi_{s}=\pi$ then the invariant distribution of this diffusion would be given by π(z)N(p;0,M). This underdamped Langevin diffusion corresponds to a continuous-time version of Hamiltonian dynamics for which the momentum component is continuously refreshed using an Ornstein–Ulhenbeck process. In this case, we can use the following integrator to discretize the diffusion equation (3.18)

3.19 $${\tilde{p}}_{t+1}∼N(hp_{t},(1-h^{2})M)\;\mathrm{and}\;(z_{t+1},p_{t+1})={\mathrm{LF}}_{t}(z_{t},{\tilde{p}}_{t+1}),$$

with h=exp⁡(−ηδ) and ${\mathrm{LF}}_{t}$ is the leapfrog integrator for $\pi_{t\delta }$. So we have forward transition kernels of the form

3.20 $$\begin{array}{rl} & m_{t+1}((z_{t},p_{t}),({\tilde{p}}_{t+1},z_{t+1},p_{t+1}))=N({\tilde{p}}_{t+1};hp_{t},(1-h^{2})M)\delta_{{\mathrm{LF}}_{t}(z_{t},{\tilde{p}}_{t+1})}(z_{t+1},p_{t+1}). \end{array}$$

We are also selecting backward transition kernels of the form

3.21 $$\begin{array}{rl} & l_{t}((z_{t+1},p_{t+1}),({\tilde{p}}_{t+1},z_{t},p_{t})=\delta_{{\mathrm{LF}}_{t}^{-1}(z_{t+1},p_{t+1})}(z_{t+1},{\tilde{p}}_{t+1})N(p_{t};h{\tilde{p}}_{t+1},(1-h^{2})M). \end{array}$$

Considering now the extended proposal and target

3.22 $$\begin{array}{rl} & \bar{q}(z_{0:T},p_{0:T},{\tilde{p}}_{1:T})=q_{\phi }(z_{0}|x)N(p_{0};0,M)\prod_{t=0}^{T-1}m_{t+1}((z_{t},p_{t}),({\tilde{p}}_{t+1},z_{t+1},p_{t+1})), \end{array}$$

and

3.23 $$\begin{array}{rl} & \bar{p}(x,z_{0:T},p_{0:T},{\tilde{p}}_{1:T})=p_{\theta }(x,z_{T})N(p_{T};0,M)\prod_{t=0}^{T-1}l_{t}((z_{t+1},p_{t+1}),({\tilde{p}}_{t+1},z_{t},p_{t})), \end{array}$$

the resulting SIS unbiased evidence estimate is given by

$$\begin{array}{rl} w_{\theta ,\phi }^{\text{sis}}(z_{0:T}) & \;=\frac{\bar{p}(x,z_{0:T},p_{0:T},{\tilde{p}}_{1:T})}{\bar{q}(z_{0:T},p_{0:T},{\tilde{p}}_{1:T})}\\ & \;=\prod_{t=0}^{T-1}\frac{\gamma_{t+1}(z_{t+1})N(p_{t+1};0,M)l_{t}((z_{t+1},p_{t+1}),({\tilde{p}}_{t+1},z_{t},p_{t}))}{\gamma_{t}(z_{t})N(p_{t};0,M)m_{t+1}((z_{t},p_{t}),({\tilde{p}}_{t+1},z_{t+1},p_{t+1}))}\\ & \;=\frac{p_{\theta }(x,z_{T})N(p_{T};0,M)}{q_{\phi }(z_{0}|x)N(p_{0};0,M)}\prod_{t=0}^{T-1}\frac{N(p_{t};0,M)}{N({\tilde{p}}_{t+1};0,M)}, \end{array}$$

where we have used the fact that as ${\mathrm{LF}}_{t}$ is a symplectic integrator, it is volume preserving and thus $|J_{{\mathrm{LF}}_{t}}(z,p)|=1$, and that $N(p_{t};0,M)N({\tilde{p}}_{t+1};hp_{t},(1-h^{2})M)=N({\tilde{p}}_{t+1};0,M)N(p_{t};h{\tilde{p}}_{t+1},(1-h^{2})M)$. As for ULA, it is possible to easily reparameterize the proposal in terms of standard Gaussian random variables. This leads to low-variance estimates of the gradients of the ELBO. Doucet *et al.* [38] provides an empirical comparison of UHA-type and ULA-type proposals in a limited Monte Carlo experiment. The performance is slightly better for UHA than for ULA, but the difference is rather small. This finding is somewhat consistent with the results of Bou-Rabee & Eberle [37], which show that the mixing time of UHA is better than that of ULA. However, we are far from having a clear theoretical guarantee of the relative advantage of using UHA compared with ULA in the context of AIS, which is very complex.

#### (iii) Optimizing backward Markov kernels

In the schemes previously described, the backward Markov kernels are selected as the reversals or approximate reversals of the forward kernels. However, it was realized early on in the literature [29] that this choice is suboptimal and the optimal backward kernels were identified. Indeed from the law of total variance, we have

3.24 $$\begin{array}{rl} {\text{var}}_{\bar{q}(z_{0:T})}[w_{\theta ,\phi }^{\text{sis}}(z_{0:T})] & \;={\text{var}}_{{\bar{q}}_{T}(z_{T})}[w_{\theta ,\phi }^{\text{mar}}(z_{T})]\\ & \;\;+E_{{\bar{q}}_{T}(z_{T})}[{\text{var}}_{\bar{q}(z_{0:T-1}|z_{t})}[w_{\theta ,\phi }^{\text{sis}}(z_{0:T})]], \end{array}$$

where $w_{\theta ,\phi }^{\text{mar}}(z_{T})$ is defined in (3.3) and the second term on the r.h.s. is null for $\bar{p}(z_{0:T-1}|z_{T},x)=\bar{q}(z_{0:T-1}|z_{t})$. So using the backward decomposition of $\bar{q}(z_{0:T})$ shows that the optimal backward kernels $l_{t}^{\text{opt}}$ satisfy

3.25 $$l_{t}^{\text{opt}}(z_{t+1},z_{t})=\frac{q_{t}(z_{t})m_{t}(z_{t},z_{t+1})}{q_{t+1}(z_{t+1})},$$

where $q_{t}(z_{t})$ is the marginal of $z_{t}$ under $\bar{q}(z_{0:T})$. While this expression is interesting, it is unclear how one could come up with an approximation of these optimal backward kernels as the marginals $q_{t}(z_{t})$ are intractable. However, inspired by recent developments in generative modelling [39], it was recently realized in [38] that it is possible to come up with sensible approximations to these optimal kernels for ULA and UHA proposals. We restrict ourselves here to ULA proposals and refer the reader to Doucet *et al.* [38] for UHA proposals. Recall that the ULA proposal we consider can be thought of as the time-discretization of the time-inhomogeneous Langevin diffusion equation (3.17). The time-reversed process $({\bar{z}}_{s})={\left(z_{T_{0}-s}\right)}_{s\in [0,T_{0}]}$ is also a diffusion given by

3.26 $$\;\text{d}{\bar{z}}_{s}=\{-\nabla \log\pi_{T_{0}-s}({\bar{z}}_{s})+2\nabla \log q_{T_{0}-s}({\bar{z}}_{s})\}\;\text{d}s+\sqrt{2}\;\text{d}{\bar{B}}_{s}\;\mathrm{and}\;{\bar{z}}_{0}∼q_{T_{0}},$$

where $\left({\bar{B}}_{s}\right)$ is another Brownian motion and $q_{s}$ denotes the law of $x_{s}$ under equation (3.17). The so-called score terms $\nabla \log q_{s}$ are intractable but this expression suggests that one should consider parameterized backward Markov transition kernels of the form

3.27 $$l_{t}(z_{t+1},z_{t})=N(z_{t};z_{t+1}-\delta \nabla \log\pi_{t+1}(z_{t+1})+2\delta s_{\phi }(t+1,z_{t+1}),2\delta I),$$

where $s_{\phi }$ is a neural network approximating the scores ${\left(\nabla q_{t}\right)}_{t=1}^{T}$. The parameters of this network can be obtained by maximizing the ELBO which coincides with a denoising score matching loss [40]. This approach can improve experimentally over the standard backward Markov kernels for both ULA and UHA proposals [38] for the estimation of the normalizing constant in these contexts. While it had been previously proposed to use backward Markov kernels of the form $l_{t}(z_{t+1},z_{t})=N(z_{t};\mu_{\phi }(z_{t+1}),\Sigma_{\phi }(z_{t+1}))$ [30,41], where the functions $\mu_{\phi },\Sigma_{\phi }$ are parameterized by neural networks, this parameterization does not exploit the structure of optimal backward kernels and performs poorly in simulations [16].

#### (iv) Combining stochastic proposals and normalizing flows

It is well known that adding some deterministic moves to the stochastic transition kernels can further improve the performance of SIS/AIS type algorithms. In practice, we simply interleave stochastic transitions with deterministic invertible transitions of the form $m_{t}(z_{t-1},z_{t})=\delta_{T_{t}(z_{t-1})}(z_{t})$ with corresponding backward kernel $l_{t-1}(z_{t},z_{t-1})=\delta_{T_{t}^{-1}(z_{t})}(z_{t-1})$. This was proposed early on by Vaikuntanathan & Jarzynski [42]; see also [43]. In [28], these deterministic moves $T_{t}$ are built using NFs whose parameters are learned by maximizing the ELBO.

#### (v) Limitations

The main limitation of the methods is that the unadjusted samplers considered here can be much more unstable than their Metropolis-adjusted counterparts. They also suffer from high memory consumption as they require storing the whole simulated Markov chain. As a result, one is limited to use short chains which limit their performance.

## 4. Further extensions

### (a) Sequential Monte Carlo

For SIS-type methods, degeneracy of importance weights typically occurs. SMC samplers, originally proposed by Del Moral *et al.* [29], introduce additional resampling, either at each step or adaptively using a non-degeneracy criterion for importance weights to mitigate this problem. The resampling has the effect of providing an unweighted cloud of particles distributed approximately according to $\pi_{t}$. Note, however, that using resampling steps for estimating normalizing constants is not always beneficial; see [29, Section 4.2.3.2]. As SMC samplers provide also an unbiased evidence of the evidence [44], they can be used to provide an ELBO. This was noted by Maddison *et al.* [19], Le *et al.* [45] and Naesseth *et al.* [46]. However, the resulting ELBO is difficult to maximize. Indeed, the resampling steps of SMC involve sampling discrete distributions. Hence, the variance of the gradient estimates is very large as we have to use REINFORCE gradient estimates, similarly to (3.14). Hence it was proposed in [19,45,46] to use biased gradient estimates which neglect the terms resorting to a REINFORCE gradient estimate. Unfortunately, as established by Corenflos *et al.* [47], this can introduce some very substantial bias. To address this problem, differentiable resampling procedures have been introduced; e.g. [47,48]. In these approaches, resampling is replaced by solving a regularized optimal transport problem. These methods perform well but are computationally quite expensive and have only been demonstrated for low-dimensional filtering problems where $d_{z}$ is moderate. In the DLVM settings where $d_{z}$ can be of the order of a hundred, it is not expected that they will fare well. One way to get around these issues is to abandon the ELBO for alternative loss functions.

In [49], the authors consider a SMC sampler combined with NFs. A NF is introduced after each resampling step to push particles towards the next target distribution. To learn these NFs, it is proposed to minimize

4.1 $$\sum_{t=1}^{T}D_{\text{KL}}[T_{t}^{\#}\pi_{t-1}||\pi_{t}],$$

where $T_{t}^{\#}\pi_{t-1}$ denotes the push-forward of $\pi_{t-1}$; see also [50]. This criterion and its gradient can be estimated using the particle approximation of $\pi_{t-1}$. A stop gradient operator is used to avoid differentiating through the SMC procedure, thus avoiding the increase in variance caused by the approximation of the differentiation through the resampling steps. Using a challenging example from lattice field theory and VAEs, the method has been shown to outperform alternatives such as the schemes proposed in [28,51]. The advantage of this method is that, unlike [19,45,46], it does not require differentiation of the SMC process.

Midgley *et al.* [52] propose an alternative approach to train a normalizing $q_{\phi }$ flow to approximate a target μ. Instead of using the mode seeking reverse KL to train the flow, one minimizes the variance of the importance weight $p_{\theta }(z|x)/q_{\phi }(z)$. This criterion and its gradient w.r.t. ϕ is approximated using a standard Metropolis-adjusted AIS sampler targeting the optimal importance distribution $\pi (z)\propto p_{\theta }{\left(z|x\right)}^{2}/q_{\phi }(z)$—an SMC sampler would also be applicable. A stop gradient operator is used to avoid differentiating through the AIS procedure.

### (b) Alternative unbiased estimates of the evidence

While most work on marginal likelihood estimation (to construct a valid ELBO) has focused on importance sampling methods and its variants, other approaches have recently been proposed. Building on the work of Rotskoff & Vanden-Eijnden [53], Thin *et al.* [54], for example, have proposed a novel unbiased estimate of evidence based on deterministic trajectories starting from points sampled by an importance distribution $q_{\phi }(z|x)$. Starting from a well-chosen transformation T (typically a conformal Hamiltonian dynamics), the estimator $w_{\theta ,\phi }^{\mathrm{neo}}(z)$ is given by

4.2 $$w_{\theta ,\phi }^{\mathrm{neo}}(z)=\sum_{k=0}^{K}\varpi_{k}(z)w_{\theta ,\phi }(T^{k}(z)),$$

where the weights $\varpi_{k}(z)=q_{\phi }(T^{k}(z)|x)J_{T^{k}}(z)/\{\sum_{i=-k}^{K-k}q_{\phi }(T^{-i}(z)|x)J_{T^{-i}}(z)\}$ ensure unbiasedness of the estimator $w_{\theta ,\phi }^{\mathrm{neo}}(z)$ when $z∼q_{\phi }(\cdot |x)$. This estimator can be easily applied with the reparameterization trick [2] and leads to a differentiable ELBO. It has been successfully used by Thin *et al.* [54] to perform inference for a variety of latent variable models.

### (c) Other related works

We have mainly focused on variational SIS based approaches compared with other classical homogeneous MCMC methods which do not provide an estimate of the evidence. Hoffman [1] considers another type of DLGM learnt with Hamiltonian Monte Carlo (HMC), by decoupling the ELBO, optimizing it in ϕ while approximating (1.2) using an HMC chain initialized at $z_{0}∼q_{\phi }(\cdot |x)$. This method, however, differs from the ones presented above, as the effective variational distribution built cannot be cast into a single objective maximization. Another related approach is the Hamiltonian VAE [55], which improves the variational family using a well-designed flow inspired by Hamiltonian dynamics. It is, however, more closely related to NFs than to MCMC samplers. Another relevant direction of work is unbiased and biased-reduced estimation of the gradient of the log-likelihood (1.2), as investigated in [56,57]. Both works extend the IWAE methodology by writing an iterated Sampling Importance Resampling scheme (i-SIR [58,59]) to re-interpret the IWAE ELBO on an extended space and use either coupled Markov chains methodology to obtain unbiased estimates of (1.2) or biased reduction techniques to improve on the IWAE’s methodology, viewing (2.5) as a self normalized importance sampling estimate.

## 5. Discussion

The marginal likelihood/evidence for deep latent variable models is intractable but low-variance and unbiased Monte Carlo estimates of the evidence can be used to define a tight ELBO. However, because we are interested in maximizing such bounds with respect to both the model parameters θ and the variational parameters ϕ, it is also necessary to have access to low-variance gradient estimates of this ELBO. Unfortunately, most standard procedures used in the statistics literature, such as AIS and SMC, provide high variance gradient estimates because they are based on accept/reject steps and/or resampling steps. To address this problem, variants of AIS based on general SIS have been proposed based on unadjusted samplers such as ULA and UHA. These provide low-variance gradient estimates through the trick of reparameterization, but at the cost of a loss of numerical stability compared with their Metropolis-adjusted counterparts. Differentiable estimators of the evidence and the ELBO have also been proposed for SMC, but these are limited to low-to-medium dimension latent variables. For high dimensional scenarios, SMC estimators of ELBO remain difficult to optimize and it may be better to use alternative criteria.

## Data Availability

This article has no additional data.

## Footnotes

1 q(u) can depend on θ,ϕ and x but this is notationally omitted.
